# The experiences of care managers and rehabilitation coordinators of a primary care intervention to promote return to work for patients with common mental disorders: a qualitative study

**DOI:** 10.1186/s12875-020-01348-x

**Published:** 2020-12-18

**Authors:** Eva-Lisa Petersson, Karin Törnbom, Dominique Hange, Shabnam Nejati, Margareta Jerlock, Carl Wikberg, Cecilia Björkelund, Irene Svenningsson

**Affiliations:** 1 Primary Health Care, School of Public Health and Community Medicine, Institute of Medicine, The Sahlgrenska Academy, University of Gothenburg, Gothenburg, Sweden; 2 Research and Development Primary Health Care, Region Västra Götaland, Göteborg, Sweden; 3 Department of Social Work, University of Gothenburg, Gothenburg, Sweden

**Keywords:** Mental disorder, Nurses, Primary health care, Rehabilitation, Sick leave

## Abstract

**Background:**

In an earlier study, PRIM-CARE RCT, a care manager implementation at the primary care centre showed improved return to work and reduced sick leave for patients with CMD. To further improve return to work, the project Co-Work-Care added a person-centered dialogue meeting between the patient, the employer and the rehabilitation coordinator, preceded by an increased collaboration between care manager, rehabilitation coordinator and GP. In this first qualitative study of the Co-Work-Care project, we explored how care managers and rehabilitation coordinators experienced the Co-Work-Care model. The purpose of this study was to explore care managers’ and rehabilitation coordinators’ perceptions and experiences of a close collaboration and the use of the person-centred dialogue meeting.

**Methods:**

From an ongoing RCT with 20 primary care centres, care managers (CMs) (*n* = 13) and rehabilitation coordinators (RCs) (*n* = 12) participated in a qualitative study with focus groups. The study was conducted in the primary health care in a Swedish region. The data was analysed with Systematic Text Condensation by Malterud.

**Results:**

Seven codes describing the participants’ experiences of the Co-Work-Care model were identified: 1) The importance of collaboration at the primary care centre, 2) Collaboration and division of roles between the RC and the CM, 3) Collaboration with the General practitioner (GP), 4) The person-centred dialogue meeting, 5) Initiating the person-centred dialogue meeting, 6) The person-centred dialogue meeting to improve collaboration with the employer, and 7) The person-centred dialogue meeting to teach about the return to work process.

**Conclusion:**

The increased collaboration within the Co-Work-Care model created a common picture and understanding of the patient’s situation. The person-centred dialogue meeting in the rehabilitation process became a bridge between the employer and the patient.

**Trial registration:**

NCT03250026 (registered August 15, 2017).

**Supplementary Information:**

The online version contains supplementary material available at 10.1186/s12875-020-01348-x.

## Background

Work is essential for the individual’s wellbeing, but also for the personal identity. In general, working people have better mental health than those who are out of work [1]. Research has shown that employment can be a protective factor against common mental disorders (CMD) [2]. Work is also important when recovering from mental health conditions [1].

In Sweden, an increasing portion of sick leave episodes are caused by CMD, i.e. depression, anxiety syndromes and stress-related disorder. Around 2% of the working population, corresponding to 50% of people on sick leave, have a CMD diagnosis as reason for absenteeism [3]. In Sweden, a majority of the individuals with CMD seek primary health care [4] as do a majority of those who have a reduced ability to work [5]. Depressive disorders impact on work ability and have extensive societal consequences [6]. The provision of a structured telephone outreach in combination with a care management programme and medication [7] appears to be one realistic way to reduce sick leave among people with CMD. The use of reality-based knowledge from the workplace in the intervention seems to reduce depressive severity symptoms [8]. Thus far, workplace interventions have not shown any considerable impact on time to return to work [9]. For professionals, one important factor to take into account is the patient’s own expectations about returning to work [10–12].

In primary health care, the Collaborative Care Model, based on Wagner’s Chronic Care Model [13], is well known. The model has four key components: team-based approach to patients, structured care plan, scheduled patient follow-ups, and enhanced inter-professional communication [14, 15]. Compared to usual care, Collaborative Care Model has been shown to be an effective way of working with patients with anxiety and/or depression [14].

In the Collaborative Care Model, different professionals such as nurses, general practitioners (GP), therapists, and paramedics collaborate with a specially trained care manager (CM) as facilitator. The CM coordinates care by maintaining a close and regular contact with patients and aligns efforts for their individual needs [16]. Studies show that a CM provides improvements both for patients with depression [17] and for the physical health of patients with chronic heart failure [18]. The use of a CM for patients with depression was evaluated in the PRIM-CARE RCT and showed positive results concerning return to work (RTW) and reduced sick leave [19].

In Sweden, a coordinator function for patients with sick leave, called rehabilitation coordinator (RC), has been implemented in the primary health care [20]. The function is mandatory and initiated by public policy [21]. The RC function provides support for patients during their sick leave and RTW process [20].

The Co-Work-Care project evolved from PRIM-CARE RCT and aims to further improve RTW and reduce sick leave time for patients with CMD. The Co-Work-Care project added a person-centered meeting between the patient, the employer and the RC, coupled with an increased collaboration between CM, RC and GP (https://clinicaltrials.gov/ct2/show/NCT03250026).

In this first qualitative study of the Co-Work-Care project, our aim was to gain knowledge of how CMs and RCs experienced the Co-Work-Care model.

The purpose of this study was to explore care managers’ and rehabilitation coordinators’ perceptions and experiences of a close collaboration and the use of the person-centred dialogue meeting, the Co-Work-Care model.

## Methods

### Design

In the present qualitative study, we used data obtained from four focus group (FG) discussions [22, 23]. Data analysis was performed according to Malterud’s [24] Systematic Text Condensation (STC). STC is a descriptive and explorative method for thematic cross-case analysis with a pragmatic approach [24].

### The co-work-care project

In the project a Co-Work-Care model was developed that included an in-depth collaboration between CM and RC, followed by a person-centred dialogue meeting between the patient and the employer with the RC serving as a dialogue moderator. In the Co-Work Care model, the process starts when the CMD patient meets the GP, who assesses the need for a sick leave certificate and refers the patient to the CM and RC. The model included an in-depth collaboration between CM and RC, based on the unique prerequisites at the PCC. During the care process and cooperation between CM, RC and GP, the patient situation becomes clearer and forms a basis for the person-centred dialogue meeting.

#### The person-centred dialogue meeting

We used the theoretical framework of the convergence dialogue meeting, a strategy that provides a way to increase RTW and reduce total sick leave time [25]. The core intervention in the convergence dialogue meeting is a dialogue between the employer and employee with the convergence dialogue meeting consultant serving as the moderator, in order to reach concrete short-term and long-term solutions [26]. The convergence dialogue meeting has been modified to meet the prerequisites of the primary care centres and thus is referred to as the person-centred dialogue meeting. This person-centred dialogue meeting is between patient and employer, with the RC serving as the dialogue moderator. The person-centred dialogue meeting is based on the patients’ perception of their situation and clarifies their needs and requirements. The aim of the meeting is to give the patient the opportunity to sit down with the employer in a calm and neutral environment, and together with the employer to describe their situation. The meeting is based on the patient’s situation and includes no negotiation. Before the meeting, the RC prepares thorough written and oral information about the aim of the meeting in order to inform the patient and the employer. The meeting is carried out in a neutral place, preferably at the primary care centres. The RC leads the person-centred dialogue meeting and is also responsible for ensuring that the patient’s needs and perception of the situation are in focus. The RC starts the meeting with the following question for the patient: “Can you tell us about how you perceive your situation?” and the following question for the employer: “How do you perceive the patient’s situation?”. The meeting continues for 45–60 min. The employer and the patient together with the RC end the meeting with a conclusion and an agreement. Finally, the RC documents in writing the facts, agreements and possible actions.

#### Care manager

A CM improves high accessibility and continuity. Health care with a CM is based on the patient’s specific needs over time and provides support regarding medical issues and self-management. It is thus a form of organisation that allows for complexity, person-centred care and interaction [19].

#### Rehabilitation coordinator

The RC provides support for the primary health care centres for effective collaboration in the sick leave certification and rehabilitation process [20]. The RC should also be a support for sick-listed patients in the sick leave process with regard to medical insurance issues. Further, the RC should coordinate rehabilitation as well as be a knowledge broker and adviser regarding certificate issues and the rehabilitation plan [20].

### Setting and participants

The participants in the present study were CMs and RCs from the intervention group at an ongoing RCT and represented urban and rural primary care centres, operated by private and public actors. The CMs and RCs underwent a one day educational session about the project Co-Work-Care. They had worked in the project for about one year. Most of the participants were nurses with an additional role as CM and/or RC. During the recurrent training sessions, all 34 CMs and RCs were invited and 25 participated in the study, including one person with both functions (CMs *n* = 13 and RCs *n* = 12). Due to lack of time, nine participants could not participate. Four focus groups (*n* = 6, n = 6, n = 6, *n* = 7) with 24 women and 1 man were selected. The participants were informed that the discussions were voluntary, audio-recorded and transcribed verbatim and kept confidential. The transcripts were not returned to the participants.

### Data collection

Focus group discussions were used to collect the data and were moderated by ELP and IS with SN (RN), MJ (PhD, RN) and CW (PhD, RN) as assessors. A guideline was developed with questions for the focus group and was presented for the participants at the focus group session. Fieldnotes were taken by the assessors during the session. The focus group discussion took place during a single session. The questions in the guideline were validated through discussions with CM, RC and within the research group. The researchers took part in the learning process during the one day educational session prior to the study start, and the researchers also shared their goal regarding the study with the participants. The focus groups took place in spring and autumn 2019 at the Primary Care Research and Development Department in Gothenburg and the duration of the focus group was 45 to 60 min. The focus group guide included the following questions: What does collaboration mean to you? How do you perceive early contacts with the employer? How do you perceive the collaboration among the CM and RC? How does this collaboration impact the communication among RC, patient and the employer? See additional file 1.

### Analysis

The analysis was conducted using the STC by Malterud [24] and was a collaboration among ELP, IS, KT, CB and DH. ELP is an occupational therapist and IS is a district nurse; both have doctoral degrees and long experience from primary health care. KT is a social worker with a doctoral degree and experience from psychotherapy in outpatient care. CB is a general practitioner and professor, and DH is a general practitioner and associate professor. Five of the researchers conducted the analyses and all were females. Malterud [24] does not use the concept of saturation concerning sampling methodology. Instead, it is important to provide coherent stories to establish an adequate information-rich sample grounded in empirical data. The analysis was data-driven, with no theoretical framework as template.

The following four steps guided the analysis: 1) The data set was read several times with an open mind to obtain an overall impression. 2) Units of meaning were identified representing different aspects of participants’ experiences of collaboration among CMs, RCs and GPs and their experiences of the person-centred dialogue meeting, coded accordingly. 3) Condensing the contents of each of the coded groups, an artificial quotation was constructed, maintaining the original terminology applied by the participants. 4) Finally, each code group was summarised to a generalised descriptions concerning collaboration and the person-centred dialogue meeting.

## Results

The close in-depth collaboration and knowledge sharing among CMs and RCs at the primary care centres created added value in the health care services. Good communication was also seen as a guarantee that all tasks were done. Participants in the study emphasised the difficulty in working with some locum tenens GPs, who often switch workplace. RCs expressed that a visit to the patient’s workplace benefited the understanding of the working environment. However, the person-centred dialogue meeting was preferably conducted at the primary care centres. There was an agreement among the participants that the RC was a bridge between the employer and the patient and that an early initiation of the person-centred dialogue meeting was beneficial for the patient. A common source of frustration was that employers generally knew so little about their own responsibilities for the working environment and for rehabilitation. The RCs perceived that the person-centred dialogue meeting sometimes revealed an unknown complex situation.

### The importance of collaboration at the primary care centres

CMs and RCs described that close collaboration and knowledge sharing among professionals at the primary care centres created added value in their health care services. The patients were ensured of receiving advice regarding RTW, irrespective of which professional they turned to. Good communication was also seen as a guarantee that all tasks were done, and that nothing fell between the cracks.

*It saves an incredible amount of time! Instead of writing a lot and sending it away, I can knock on the door and leave the information directly to whom it concerns.* (FG 1)

Sharing different views on how best to treat patients with CMD was said to improve the quality of health care. However, it was stressed that to make this collaboration work, professionals needed to take a personal responsibility and be willing to lose prestige for the good of the patients. Problems with cooperation were lifted as something that could harm the patient’s recovery or even increase their emotional suffering.

### Collaboration and division of roles between the rehabilitation coordinator and the care manager

CMs and RCs thought that the Co-Work-Care model had improved and developed the collaboration at the primary care centres. Through this method, the CM and the RC had further developed a common working model for patients with CMD. A goal was to be flexible about patients’ needs, so that patients received help from the most suitable professional at the right time. In most cases the CM took the role to first understand the patient’s situation, and then to thoroughly communicate this information to the RC. The role of the RC was to use the information strategically, in a way that benefitted the patient during the person-centred dialogue meeting with the employer. The main goals in this meeting were to create a common ground concerning the patient’s situation and to support the patient in relation to the employer. The RC also had the main role in practical matters concerning the sick leave.

*I can feel that collaboration is like putting an equal sign between collaboration and qualitative-enhancing measures. … .. to get more substantiated decisions.* (FG 4)

### Collaboration with the GP

According to the participants, the CM and the RC planned the interventions for patients with CMD, while the GPs prescribed medication or were involved in practical matters regarding sick leave. Participants perceived that the GPs often followed their recommendations, since they were expected to have extensive information about the patients’ situation.

*The GPs benefit so much from a close cooperation with us. I have spoken many hours with the patient, and then I summarise everything in notes. I have the habit of reminding the GPs to read what I have written. You have to make sure and work a little harder to remind new GPs to read the notes.* (FG2)

Participants emphasised the difficulty posed by the fact that many GPs switched positions often, and did not have time to become familiar with the working model for patients with CMD. Participants also perceived that, at a structural level, GPs were not expected to be very aware of the patient’s overall situation. Both these issues were seen as barriers to a more profound collaboration with the GPs in general.

### The person-centred dialogue meeting

The main purpose of the person-centred dialogue meeting was to create a basis for collaboration where the patient felt safe to communicate needs and concerns from his/her own perspective. The primary care centres was seen as a neutral place for the meeting, whereas the workplace was considered as potentially intimidating from a patient’s point of view.

Participants agreed that the RC should function as a bridge between the employer and the patient, to lay the basis for a healthy relationship that could contribute to a sustainable RTW. A common experience was that when the employer and the patient tried to solve problems entirely without help from the RC, they often fell into habitual relationship patterns which complicated their ability to find sustainable solutions.

*It is generally beneficial to include an external part at the meeting. The employer is higher in the hierarchy, and the patient is in a vulnerable situation. Therefore, the patient may benefit from getting support from someone who is external.* (FG 1)

The person-centred dialogue meeting was emphasised as crucial when dealing with an unsustainable work situation. RCs described how they made sure to clarify and confirm perspectives of the patient as well as the employer, in order to create a plan based on a common understanding.

Several RCs wanted the person-centred dialogue meeting also to function as a platform for making a rehabilitation plan. They considered it ineffective to first gather a lot of information about the patient, and then not be able to use this in a purposeful way during the meeting. Others felt that it was better to keep the person-centred dialogue meeting as an initial “ice-breaker” discussion, to create a solid ground upon which to build further work.

In some cases, participants received information of a personal nature from the patients that could have been meaningful for understanding the development of CMD and for the caring process. Thus, RCs needed to know which information they could share during the person-centred dialogue meeting. Nevertheless, it was mentioned that even when patients experienced problems outside of work, their overall situation could be improved by creating a healthier work situation.

*… so that the contact with the workplace becomes a health factor, so that the patient can get better, and not get into isolation or social exclusion. To create this, I don’t see the underlying problem as especially important … what exactly the problem is, so to speak*. (FG 3)

### Initiating the person-centred dialogue meeting

Having a meeting early in the sick leave period to create a plan with the patient and the employer was considered beneficial for the patient. A shared experience was that when the RC scheduled the person-centred dialogue meeting at a later stage, it became more difficult for the patient and the employer to reach a shared view of the problems.

RCs experienced that patients often interpreted employer’s unwillingness to reach out as indifference, while the employer could be concerned about not putting a pressure on the patient. To avoid such misunderstandings, an early meeting could serve as an icebreaker, sparing both parties some distress, and making it easier for the patient to focus on getting well.

*It is also easier when the patients still have their work identity. If you wait for too long, they will more and more identify themselves as being sick.* (FG 3)

Scheduling a first talk with the employer early on also meant practical advantages concerning sick leave. The RC could then form a time plan together with the patient and the employer, so that all parties knew what to expect and how to act, well before the sick leave ended.

*Some changes that need to be done cannot be fixed within a day. Sometimes the employer needs to start up a process at the workplace, and therefore we need to start this communication early on.* (FG 4)

Having a collaborative talk with the employer prior to the person-centred dialogue meeting was done to prepare oneself for how the situation was perceived by the employer and thus facilitated the development of a return to work plan. However, participants explained that if the patient’s condition was too poor, they had to wait with the person-centred dialogue meeting, to avoid the risk of not being able to establish a functioning collaboration between the employer and the patient.

### Person-centred dialogue meeting to improve collaboration with the employer

RCs perceived employers as being generally humble about their lack of knowledge of mental health disorders, and they seemed grateful about receiving practical help and valuable advice from the primary care centres. RCs communicated a frustration about the fact that employers generally knew so little about their own responsibilities for working environments and rehabilitation. Some participants even thought that patients would not have become ill in the first place if employers had known more about these issues.

*As a starting point, I usually ask the employer if they have easier, uncomplicated tasks that we can offer the patient. It is often difficult for the patients to concentrate on several tasks simultaneously.* (FG 3)

Participants agreed that having full cooperation with the employer was crucial in order to help them change possibly harmful working conditions. It was also agreed that if nothing changed, the risk for the employee to end up on sick leave again was high.

### Person-centred dialogue meeting to teach about the return to work process

Collaboration was also considered important to resolve misunderstandings about how to think about the RTW process. Participants emphasised that returning to work had to be seen as an ongoing, one-step-at-a-time process.

*Some people think that you need to feel completely well before you can return to work. It’s more about that you get well by gradually increasing the workload.*

(FG 3)

The way to a better working situation was said to go through creating a healthier and sustainable working situation. However, it was mentioned that at the beginning of a collaboration, both employers and patients could share the idea that resting at home was the best way to recover one’s working capacity. Finding ways to collaborate with the employer, creating solutions for better working conditions, and planning for a gradual return to work were therefore considered incredibly important.

*During the person-centred dialogue meeting, employers often understand things that they haven’t seen before or understood the extent of. The conversations can work as an eye-opener for the patient’s situation and for the working environment.* (FG 2)

According to participants, the person-centred dialogue meeting sometimes revealed a complex situation, for example that the patient had been bullied at work or had an overly complicated relationship with the employer. In some cases, participants decided that problems were so difficult to resolve, that it would be in the patient’s best interest to change workplace.

## Discussion

The purpose of this study was to explore care managers’ and rehabilitation coordinators’ perception and experiences of a close collaboration and the use of the person-centred dialogue meeting, i.e. the Co-Work-Care model.

In the Co-Work-Care model, the CM and RC have different roles that complement each other. The CM gives support to the patient in medical issues and self-management, and the RC supports the patient on sick leave during the rehabilitation process until return to work. Both CM and RC and the close in-depth collaboration and knowledge sharing created an added value in the understanding of the rehabilitation process. A good communication and a common working model (the Co-Work-Care model) were seen as a guarantee to reach the best solution for the patient, which often entailed an early introduction of the person-centred dialogue meeting and gradual RTW. Both RCs and CMs stressed the difficulty when working with some GPs with temporary positions, as this hampered continuity of care and prevented them from being able to become familiar with the working model for patients with CMD. The RC became a bridge between the employer and the patient, and an early person-centred dialogue meeting was perceived beneficial for the patient. The RCs experienced frustration when they realised that employers lacked knowledge of their responsibilities for the work environment and the rehabilitation process. The RC perceived that the person-centred dialogue meeting sometimes revealed unknown complex situations.

The close collaboration and knowledge sharing among the professionals seem to fit well with the Collaborative Care Model [27]. We believe that the in-depth collaboration between CM and RC added a new, extended knowledge about each other’s working areas, giving a further value to the patient. However, the study does not reveal how the actual collaboration actually was carried out. To achieve a successful working collaboration process, the CM and RC need to be willing to collaborate, and the organisation needs to provide the opportunity for such collaboration [28, 29]. The professional’s individual background is also important when defining the patient’s need for RTW, according to Eikenaar [30]. That the CM and RC shared goals is important, in order to avoid that the patient receives different advice from different professionals [31–33]. However, de Riijk et al. showed that the professionals’ dependency on each other in achieving one’s goal was more important for cooperation than the goal as such [28]. Good communication between the professionals can become a safety net that prevents the patient from falling between the cracks [27, 34]. Thus, collaboration within the team, where every professional contributes with different competencies, is crucial to gaining a picture of the patient’s entire situation. The Co-Work-Care model with its focus on the collaboration and person-centred dialogue meeting appears to have an important impact on both CMs’ and RCs’ way of working in the health care and the RTW process. The professionals became flexible about the patient’s needs, meaning that the most suitable professional was engaged at the right time.

The participants perceived that GPs often followed the CMs’ and RCs’ recommendations, since they were expected to have extensive information about the patient’s situation. When this collaboration worked well, it benefitted the patient. However, when locum GPs were involved, there was not enough time for these GPs to be a part of the Co-Work-Care model for the patients.

It seems preferable to accomplish the person-centred dialogue meeting at the primary care centre, a neutral location, instead of at the workplace, which might be intimidating for the patient. A common experience among the RCs was that the patient and the employer often fell into a habitual relationship pattern that complicated a sustainable solution. In order to create a plan for a common understanding, it was important to clarify and confirm the perspectives of the patient as well as of the employer. Some RCs wanted the person-centred dialogue meeting to be a platform for making the rehabilitation plan, while others wanted it to be an initial icebreaker to create a solid ground upon which further work could be built. However, the main intention of the person-centred dialogue meeting is to give the patients an opportunity to put into words how they perceive their entire situation. We believe that when the patients express their views about the situation, in presence of the employer and with the support of the RC, this creates an opportunity to gain a common picture and understanding of the patient’s situation [35]. To encourage and support the patient to remain in or (re)-enter work as soon as possible is crucial in the RTW process [1].

Both RC and CM perceived that an early person-centred dialogue meeting involving the employer was important for the patient’s RTW. This is in line with Hiske et al., [36] who showed that work-related interventions that support workers with depression improve work ability. A common view of the patient’s situation is important to be able to intervene and make predictions about the sick leave process.

The RCs stressed that the employers generally were humble about their lack of knowledge about work-related mental health conditions. The collaboration with the employer in order to give practical help and valuable advice was therefore considered vital. Further, the importance of work-related interventions, the severity of symptoms, and the individual expectations all need to be highlighted [37] and to be taken seriously in the person-centred dialogue meeting.

The person-centred dialogue meeting sometimes revealed a complex situation, and complex situations cannot be solved with simple models. A flexible approach is necessary when new collaboration methods are implemented. In the present study, the RC was able to support the collaboration between the employer and the employee in order to find solutions for better working conditions, an aspect that was highlighted as important. The collaboration between the employer and the employee is vital in the RTW, according to Hoefsmit [38]. The Co-Work-Care model gives opportunities to strengthen the patient’s courage to talk about the entire situation and to understand the different components in the patient’s working situation. The importance of understanding the components included in the patient’s entire situation has also been shown in an early study [35].

We believe that these results could be transferable to professionals supporting other patient groups not only in primary health care in Sweden, but also in other countries.

### Strengths and limitations

We used four focus groups with 25 individuals in all. The number of participating individuals was a strength in the study. The focus group discussions allowed the participants to reveal to each other their experiences of working as CMs and RCs and to share their perceptions about the Co-Work-Care model. The collected data was rich and varied with many different statements. The collaborative work during the analytical phase incorporated different professionals’ points of view, which broadened the perspective.

A potential weakness in the study is that two of the co-authors, ELP and IS, both collected data and took part in data analysis. This potential bias was compensated for in the analysis by the participation of the other co-authors (DH, KT, CB) in the analysis. Another potential weakness could be that it might be difficult to bring up sensitive experiences in a group discussion. We did not include GPs in the focus group discussions, but plan to perform focus group discussions also with GPs.

## Conclusion

The increased collaboration between CM and RC created an opportunity to gain a common picture and understanding of the patient’s situation. The person-centred dialogue meeting led by the RC became a bridge between the employer and the patient. The Co-Work-Care model seems to be beneficial also for the person with CMD.

However, the locum GPs’ temporary employment situation led to a reduction of continuity and a lack of collaboration within the team. This issue was sometimes seen as a barrier to a more profound collaboration with the GPs in general.

The participants demonstrate the value of a cohesive approach where collaboration is the glue between the professionals and the person-centered dialogue meeting revealed the patients situation.

## Supplementary Information

**Additional file 1.** The focus group guide.

## Data Availability

To protect the participants confidentiality, the data sets generated and analysed during the current study are not publicly available due to Swedish law, but are available from the corresponding author upon reasonable request.

## Abbreviations

CM: Care manager
CMD: Common mental disorder
GP: General practitioner
RC: Rehabilitation coordinator
RTW: Return to work
