# Supplementary material for: The experiences of care managers and rehabilitation coordinators of a primary care intervention to promote return to work for patients with common mental disorders: a qualitative study
Source: BMC Fam Pract. 2020 Dec 18;21:272. doi: 10.1186/s12875-020-01348-x (PMC7749497; doi:10.1186/s12875-020-01348-x)
Supplement: Supplementary file 1 — Additional file 1. The focus group guide. [file 12875_2020_1348_MOESM1_ESM.docx]

**Additional file 1**

**The focus group guide:**

What does collaboration mean to you?

How do you perceive early contacts with the employer?

How do you perceive the collaboration among the CM and RC?

How does this collaboration impact the communication among RC, patient and the employer?
